# Distinctive Features of the Human Marginal Zone and Cajal–Retzius Cells: Comparison of Morphological and Immunocytochemical Features at Midgestation

**DOI:** 10.3389/fnana.2016.00026

**Published:** 2016-03-18

**Authors:** Lyubov A. Tkachenko, Pavel A. Zykin, Ruslan A. Nasyrov, Elena I. Krasnoshchekova

**Affiliations:** ^1^Laboratory of Functional Neuromorphology, Department of Cytology and Histology, Saint-Petersburg State UniversitySaint-Petersburg, Russia; ^2^Department of Pathological Anatomy, Saint-Petersburg State Pediatric Medical UniversitySaint-Petersburg, Russia

**Keywords:** cortical development, marginal zone, Cajal–Retzius cells, neurofilaments, reelin, human fetal brain

## Abstract

Despite a long history of research of cortical marginal zone (MZ) organization and development, a number of issues remain unresolved. One particular issue is the problem of Cajal–Retzius cells (C–R) identification. It is currently based on morphology and Reelin expression. The aim of this research is to investigate MZ cytoarchitectonics and Reelin-producing cells morphotypes in the superior temporal, pre- and postcentral cortex at GW24–26. We used Reelin (Reln) as the marker for C–R cells and microtubule-associated protein 2 (MAP2) and neurofilament heavy chain protein (N200) as markers of neuronal maturation. The MZ of all of the investigated areas had the distinct cytoarchitectonic of alternating cell sparse (MZP, SR) and cell dense (SGL, DGL) layers. The distribution of the neuromarkers across the MZ also showed layer specificity. MAP2-positive cells were only found in the SGL. N200 and Reelin-positive neurons in the MZP. N200-positive processes were forming a plexus at the DGL level. All of the N200-positive neurons found were in the MZP and had distinctive morphological features of C–R cells. All of the N200-positive neurons in MZ were also positive for Reelin, whereas MAP2-positive cells lack Reelin. Thus, the joint use of two immunomarkers allowed us to discern the C–R cells based on their morphotype and neurochemistry and indicate that the Reelin-positive cells of MZ at 24–26 GW were morphologically C–R cells. In the current study, we identified three C–R cells morphotypes. Using a 3D reconstruction, we made sure that all of them belonged to the single morphotype of triangular C–R cells. This approach will allow future studies to separate C–R cells from other Reelin-producing neurons which appear at later corticogenesis stages. In addition, our findings support the assumption that a plexus could be formed not only with C–R cells processes but also possibly by other cell processes by the poorly researched DGL, which is only allocated as a part of the human MZ.

## Introduction

The marginal zone (MZ) of the cortex is the part of the early preplate that remains above the cortical plate (CP). Its neurons, Cajal–Retzius cells (C–R cells), have a key role in migration and differentiation of CP neurons during prenatal development (Bystron et al., 2008). In spite of the many studies performed since the original descriptions of Cajal (1891) and Retzius (1893) it is surprising that the problem of C–R cell identification that is based on both their morphology and marker protein expression is still unresolved. In the last decade a growing number of reviews have been devoted to this problem (Soriano and del Río, 2005; Meyer, 2010; Gil et al., 2014; Kirischuk et al., 2014; Martínez-Cerdeño and Noctor, 2014; Marín-Padilla, 2015). Despite such steadily growing interest, the morphological characteristics of C–R cells during corticogenesis are not sufficiently defined.

During early stages of prenatal development, the morphology of C–R cells in different species (lizard, mouse, rat, cat, monkey and human) are similar and could be described as mono- or bipolar horizontally oriented cells localized in close vicinity to the pial surface of the cortex. Later, for different animals, the peak of morphological diversity and quantity of C–R cells occur at different developmental stages. First, C–R cells in mice and rats appear at the 10–11th embryonic day (E10–11) and peak in morphological complexity and diversity at the 4–7th postnatal day (P4–7; Radnikow et al., 2002; Hevner et al., 2003; Cabrera-Socorro et al., 2007; Anstötz et al., 2014; Ma et al., 2014). Their classification is based on dendrite number: “typical” C–R cells have one horizontal dendrite and “atypical” have multiple dendrites (Radnikow et al., 2002; Ma et al., 2014).

In the primate (macaque) brain, C–R cells first appear on E38 and in humans on 5–6 gestational week (GW 5–6; Meyer et al., 2000; Zečević and Rakic, 2001; Rakic and Zečević, 2003; Bystron et al., 2008). Peak in morphological complexity is observed at midgestation: E89 for macaque and GW 24–28 for humans (Zečević and Rakic, 2001; Kostović et al., 2004). At this stage C–R cells establish an extensive population with more elaborate dendrite branching compared to rodents. In humans it is customary to distinguish three types of C–R cells: (1) pyriform, triangular, or monopolar type of Cajal and Retzius; (2) so-called horizontal or bipolar type of Cajal and Retzius; and (3) so-called irregular or stellate type of Cajal (Marin-Padilla and Marin-Padilla, 1982; Marín-Padilla, 2015). Marín-Padilla (2015) proposes that those cells could, in fact, be just one type of cell dissected in different orientations. The rationale in favor of this hypothesis features of axonal branching, which predominantly descends and give collaterals in middle, but not in the deep part of the layer. In the MZ of primates and humans, these axons form a dense plexus and do not go deeper (Meyer, 2010; Marín-Padilla, 2015). In rodents such a plexus does not form (Derer et al., 2001). According to the majority of researchers, the plexus is a distinctive morphological feature of primate and human MZs (Zečević and Rakic, 2001; Meyer, 2010; Marín-Padilla, 2015).

Since the discovery of the ability of C–R cells to produce Reelin, this protein has become their neurochemical marker (Ogawa et al., 1995). A Reelin signaling pathway was found to be necessary for layer-wise positioning of migrating cells in CP. Its malfunction inevitably leads to deviations from normal radial migration of projection neurons (see D’Arcangelo, 2014). In humans two types of Reelin-producing cells have been found in the MZ: large C–R cells and small C–R-like cells found under the subpial granular layer (SGL; Meyer and Goffinet, 1998). This group of researchers showed that small C–R-like cells were generated later than the large C–R cells and that they, supposedly, differentiate from the SGL’s granular cells (Meyer et al., 1999, 2002). Such cells were described in the cortex of primates at the later stages of prenatal development. In contrast to C–R cells, these cells were GABAergic neurons (Zečević and Rakic, 2001). Postnatally, in both animals and humans, a set of GABAergic interneurons producing Reelin was found within layer I (Deguchi et al., 2003; Miyoshi et al., 2010; DeFelipe et al., 2013; Ma et al., 2014).

By now, it is clear that the current use of the term “C–R cells” for any Reelin-producing cell does not exclusively include classical C–R cells. In addition, the morphology and quantity of classical C–R cells and cells producing Reelin change during development. To match C–R cells with Reelin-producing cells, there is a need to combine morphological and cytochemical approaches. However, such research is not currently available.

During ontogenesis, not only the quantity and morphological diversity of C–R cells changes, but the cytoarchitectonics of the MZ also undergo dramatic alternations. The MZ is the most complexly organized area in prenatal brain of primates and human with up to six distinct sublayers (Zečević and Rakic, 2001; Kostović et al., 2004; Judaš and Pletikos, 2010). In the human MZ at GW 18–28, six sublayers have been distinguished: (1) cell-poor marginal stripe (Randstreifen); (2) subpial granular layer (SGL); (3) marginal zone proper (MZP); (4) stratum lucidum (SL); (5) deep granular layer (DGL); and (6) stratum radiatum (SR; Kostović et al., 2004). Some of the sublayers are transitory and can only be found at specific gestational periods. The SGL, for instance, first appears at GW 11–15 and vanishes at GW 27–35 (Zečević and Milosevic, 1997; Zečević et al., 1999; Judaš and Pletikos, 2010). The other transient layers are also characterized by certain developmental periods. The SL can be observed from GW 20 to 28 and the DGL and SL from GW 15 to 34 (Kostović et al., 2004).

It is obvious that the MZ cytoarchitectonics and neuronal composition depend on CP neuron differentiation because the apical dendrite of pyramidal cells reaches and branches out in layer I. Starting from the second gestational trimester, cerebral cortex development in humans occurs heterochronically, making it difficult to research C–R cell development. For the first time, the heterochrony of cerebral cortex development was shown in the middle of the last century in the course of detailed histological studies (Poliakov, 1937, 1966). Contemporary studies based on MRIs confirm the previous studies (Rajagopalan et al., 2011; Zhang et al., 2013). Notwithstanding differences in approaches, the research results indicate that the cortex around the Sylvian fissure (superior temporal, parietal, frontal) leads in development compared to other areas. Our previous research on human neocortex development during the second gestational trimester also agrees with this conclusion. Using immunocytochemistry against microtubule associated protein 2 (MAP2), it was shown that the first differentiated neurons appear in layer eV of the superior temporal cortex, posterior frontal and anterior parietal lobes at GW 18. Up to GW 25–26, their number increases. In other cortical areas at that age, no such cells were identified (Krasnoshchekova et al., 2010).

Thus, the current knowledge of MZ organization, morphological and neurochemical features of C–R cells in the human neocortex indicate high C–R cell polymorphism, which reaches its greatest diversity at the end of the second trimester. C–R cells distinctively express Reelin, but it should be noted that not all Reelin producing cells are, in fact, C–R cells. To date the distinctive features of C–R cells processes were not used for identification of Reelin positive cells. MZ organization is directly dependent on the ongoing developmental processes in CP. Available studies of MZ do not take developmental heterochrony of CP into consideration, using only gestational age of a fetus. At the same time the spatiotemporal characteristics of CP development in different areas is likely to correlate with the differentiation of cortical pyramidal neurons in different areas of the plate in fetuses of the same gestational age. The CP heterochrony of development during the prenatal period should also be taken into consideration. The aim of this research is to investigate MZ cytoarchitectonics and C–R cell identification that is based on their morphology and Reelin expression in areas which are at the same stage of pyramidal neurons differentiation during GW 24–26.

## Materials and Methods

The brains of 10 human fetuses aged GW 24–26 were obtained from legal autopsies in Saint-Petersburg State Pediatric Medical University, according to national guidelines with the approval of the Ethics Committee of the Saint-Petersburg State University (Health and Human Services (HHS) IRB 00003875). Brains without apparent neuropathological alteration were fixed in 4% buffered para-formaldehyde. Postmortem delays did not exceed 24 h. After fixation one hemisphere of each brain was embedded in formalin fixed paraffin embedded (FFPE) and cut in a series of 15-μm-thick sections in the coronal plane. Blocks of superior temporal, pre- and postcentral gyrus of the neocortex from the other hemisphere were cryoprotected in 20% sucrose and cut with a cryostat (Leica CM — 3050S, Leica Biosystems Nussloch GmbH, Nussloch, Germany) to 70-μm-thick sections in coronal planes.

We used the following neuromarkers: Reelin (Reln) as a marker for C–R cells and MAP2 and neurofilament heavy chain protein (N200) as markers of neuronal maturation to label differentiated and mature neurons, respectively. Furthermore, N200 was also used to separate C–R cells from other Reelin-expressing neurons because there is evidence that antibodies against medium and heavy neurofilament subunits are specific markers of C–R neurons in the human neocortical MZ (Verney and Derer, 1995).

### Single and Double Immunofluorescence

Sections were pretreated for antigen retrieval in a microwave oven (700 W, 5 min) in Tris-buffered saline (TBS) pH 7.0 or citrate buffer pH 6.0. Blocking solution contained TBS pH 7.4 with 10% normal goat serum (Sigma, cat#G9023) and 0.1% Triton X-100 (Sigma, cat#93443). They were applied for 2 h at room temperature. Sections were incubated with primary antibodies in a humid chamber at 4°C overnight. The following primary antibodies were used for single, double or sequential double immunofluorescence: mouse anti-reelin (Reln, 1:250, Millipore Cat# MAB5366 RRID:AB_2285132), mouse anti-neurofilament 200 (N200, 1:250, Sigma-Aldrich Cat# N0142 RRID:AB_477257), rabbit anti-N200 (1:250, Sigma-Aldrich Cat# N4142 RRID:AB_477272), mouse anti-microtubule-associated protein 2 (MAP2, 1:200, Sigma-Aldrich Cat# M9942 RRID:AB_477256). The primary antibodies were detected by a 2-h incubation at 37°C with corresponding secondary antibodies diluted 1:250: rabbit anti-mouse IgG, conjugated to Alexa Fluor 488 (Life Technologies Cat# A11029 RRID:AB_10566286) or Alexa Fluor 568 (Invitrogen, cat#A11004) and goat anti-rabbit IgG, conjugated to Alexa Fluor 488 (Life Technologies Cat# A11008 RRID:AB_10563748) or Alexa Fluor 568 (Life Technologies Cat# A11011 RRID:AB_10584650). In case of double labeling, a cocktail of primary antibodies followed by a cocktail of secondary antibodies was used. Nuclei were stained with 4′,6-diamidino-2-phenylindole (DAPI). Sections were mounted in PBS-glycerol. A control for nonspecific staining was performed by replacing a primary antibody with normal serum from the species in which the secondary antibodies were risen. Sections were analyzed with the fluorescence microscope Leica DM5500 and scanning confocal microscope Leica TCS SP5.

### Sequential Double Immunofluorescence

For a double labeling with Reln and N200 that was raised in the same species, a sequential immunostaining method was used (Tornehave et al., 2000). Sections were analyzed and captured between cycles of immunostaining for Reln and N200. To prevent cross-talk between fluorochromes, the first round of staining was performed using Alexa-568 and the second with Alexa-488. To compare the distribution of cytochemical and cytoarchitectural organization of the MZ, the same sections were processed for Nissl staining after double sequential immunostaining. To avoid differences in the degree of tissue shrinking between the Nissl stain and immunofluorescence, slides were dehydrated in graded Ethanol before immunostaining.

### Morphometry, Reconstruction and Statistical Data Analysis

Morphometry of cortical layers and neurons of MZ was determined on captured digital images of sections using a FIJI (Fiji, RRID:SciRes_000137, SCR_002285) image processing package (Schindelin et al., 2012).

A gray level index (GLI) was used to show inter-laminar differences. It shows the density of cell bodies and their sizes per volume unit. Selected locations on Nissl-stained sections, which were not distorted by cortical folding, were used to generate a vertical profile using the FIJI “plot profile” function. A profile was built throughout the cortical depth, starting at the pial surface and ending at the subplate (SP).

Areas of MAP2-positive neuron soma in the CP were measured on (20) FFPE sections of the precentral gyrus.

The number of immunolabeled cells in the MZ was assessed on frozen sections stained for N200 with monoclonal (mN200) and polyclonal (pN200) antibodies at adjacent sections. Twenty neighboring optical fields of view were selected in 30 slices of the precentral cortex and examined at 200× magnification. The total number of immunolabeled cells was counted.

The morphology of C–R cells and the underlying plexus was assessed on frozen sections. Cells and processes were reconstructed from confocal stacks using “Simple Neurite Tracer” (Longair et al., 2011) from a FIJI package (Schindelin et al., 2012) and Bitplane Imaris 8.1 (Imaris, RRID:nif-0000–00314, SCR_007370). We measured the following morphological parameters: area of cell soma; length of ascending and descending processes; and their diameter.

All data were presented as the mean ± SEM. A Mann-Whitney *U*-test with a *P*-value < 0.01 was used for statistical analysis.

## Results

### Marginal Zone Organization at Midgestation

Human neocortex development shows pronounced heterochrony. It is not enough to take into consideration only gestational age. Thus, an MZ organization study was carried out on the cortical areas undergoing the same stage of differentiation. The quantity and localization of pyramidal neurons correlate with the neuronal composition of the MZ and could be used as an important index of differentiation of given area at given time.

The cytoarchitectonics and differentiation of the pyramidal neurons were assessed by the MAP2 immunofluorescence on the FFPE slices. At GW 24–26, the superior temporal, pre- and postcentral cortex were similar in terms of localization in the CP and morphology of MAP2-positive cells.

**Figure 1:**
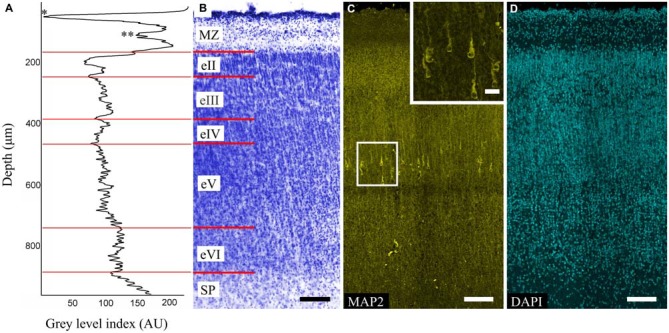
**Organization of the precentral gyrus at gestational week (GW) 26. (A)** Gray level index (GLI) of the cortex layers (*) subpial granular layer (SGL) of marginal zone (MZ). (**) Deep granular layer (DGL) of MZ. The *x*-axis represents the relative cortical depth of a selected zone in μm, and the *y*-axis represents the GLI values in arbitrary units in a range from 0 to 255. **(B)** Photomicrograph of a Nissl-stained paraffin section. Roman numerals indicate the layers of the cortical plate (CP). The red lines indicate the borders of each layer. **(C)** Photomicrograph of precentral gyrus FFPE section stained for microtubule-associated protein 2 (MAP2). Note the MAP2-positive pyramidal cells in layer eV. Box shows the position of the area given at a higher magnification in insert **(D)** 4′,6-diamidino-2-phenylindole (DAPI) staining for anatomical reference. Scale bars: **(B–D)** 100 μm, insert in **(C)** 20 μm.

The MZ, CP and SP were defined for each area. The CP was poorly stratified and characterized by alternating dense (eII, eIV, eVI) and sparse (eIII, eV) cell layers (Figures 1B,D). This alteration was also evident on the GLI graphs (Figure 1A).

The MAP2-positive cells found in the CP of all of the investigated areas were pyramidal cells and localized only in layer eV (Figure 1C). The cells were separated into three categories based on their size: small (45–90 μm^2^), medium (91–190 μm^2^), large (191–380 μm^2^). Of all cells in layer eV, 21% and 72% were small and medium cells respectively and were evenly dispersed in the layer, whereas the large ones (7%) occupied only its deep part. The apical dendrites could be traced up to layer eII. Some of the largest cells found in the precentral gyrus apical dendrite bifurcation on the threshold of the MZ could be observed. Basal dendrites were clearly defined, but only the proximal parts with 1–2 branches could be traced. The position of the cells in layer eV was refined by the GLI on the adjacent Nissl-stained sections (Figures 1A,B).

**Figure 2:**
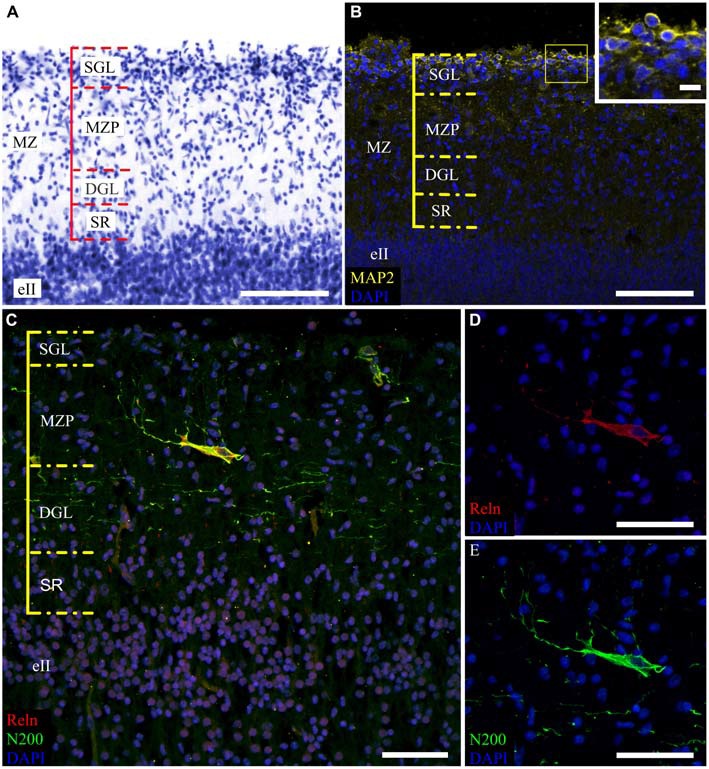
**Cytoarchitectonics and features of the MZ neurons in the fetal superior temporal gyrus at GW 26. (A)** Microphotograph of Nissl-stained paraffin section. Note the MZ cytoarchitectonics. **(B)** Photomicrograph of paraffin section stained for MAP2, SGL MAP2-positive cells is shown in the insert. **(C)** C–R cell of the same area double stained for Reelin **(C,D)** and neurofilament heavy chain protein (N200) **(C,E)**. Note the N200-positive plexus. Scale bars: **(A–C)** 100 μm; **(D,E)** 50 μm, insert in **(B)** 10 μm.

The MZ had a clear border with the CP. Some sublayers could be defined based on the cell density. Directly below the pia matter was the thin cell-dense SGL. Under this was a sparse cell layer called the MZP. Beneath this was the deep granular layer (DGL), which has more cells than MZP. Between the DGL and eII was the SR with rare scattered cells (Figure 2A). The designations of the sublayers were given based on Kostović et al. (2004).

The distribution of neuromarkers (MAP2, N200, Reln) across the MZ also showed layer specificity. Small, tightly packed MAP2-positive cells were located in the SGL (Figure 2B). Double labeling experiments with pN200 antibodies and Reelin reveal that 20% of the cells were positive to both markers, while the rest were positive only to Reelin. Experiments with Reelin and mN200 antibodies showed total (100%) co-localization. Oval, triangular, fusiform N200-positive cells were positioned deeper in the MZP. Their processes had a predominantly horizontal orientation parallel to the pial surface. The manner of their branching, as well as Reelin positivity, allowed us to classify them as C–R cells (Figures 2C–E). Below the MZP, there was a plexus of N200-positive horizontal fibers (Figure 2C). The zone between the plexus and eII was devoid of MAP2-, N200- or Reelin-positive elements. Because the immunofluorescence with N200 yielded a fine Golgi-like stain and because the FFPE sections were too thin to reveal the whole cell body and stem processes, further studies of the MZ were performed on the thick frozen sections from the same areas of the other hemisphere.

### Morphological Features of Reelin-Producing Neurons

**Figure 3:**
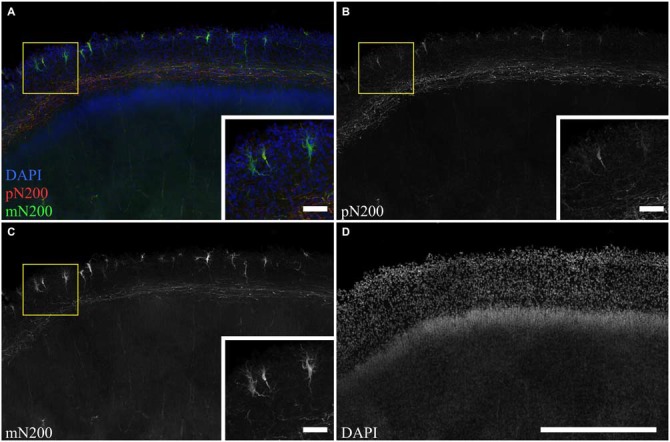
**The difference in C–R cells and plexus immunostaning in the MZ for poly- and monoclonal anti-N200 antibodies. (A–D)** Photomicrograph of slice stained with pN200 (red), mN200 (green), DAPI (blue). Box shows the position of the area given at a higher magnification in inserts. Scale bars: **(D)** 500 μm; inserts in **(A–C)** 50 μm.

To determine whole population of C–R cells and assess their morphological features, two primary antibodies for N200 were used: monoclonal (mN200) and polyclonal (pN200), along with an antibody against Reelin. Depending on the type of antibody against N200, different quantities of positive cells were visible (Figure 3). The subsequent slices of the precentral sulcus were treated with mN200 and pN200. The mean number of visible cells in 10000 μm of the MZ for mN200 was 73 ± 18 and for pN200, 15 ± 6 cells. Moreover, the polyclonal antibodies were less selective and had higher background, even with a prolonged blocking step. Thus, only the results with mN200 are reported in this article.

Using subsequent slices for single labeling, it was shown that Reelin and N200 was expressed in the same cells, but in different compartments. Reelin was mostly found in the pericarion, while N200 was found in the body and processes. Because both antibodies (Reln and mN200) were raised in mouse, the sequential immunostaining method (Tornehave et al., 2000) was used. The first round of staining used Reln with an Alexa-568 labeled secondary antibody, then mN200 with an Alexa-488 labeled secondary antibody.

**Figure 4:**
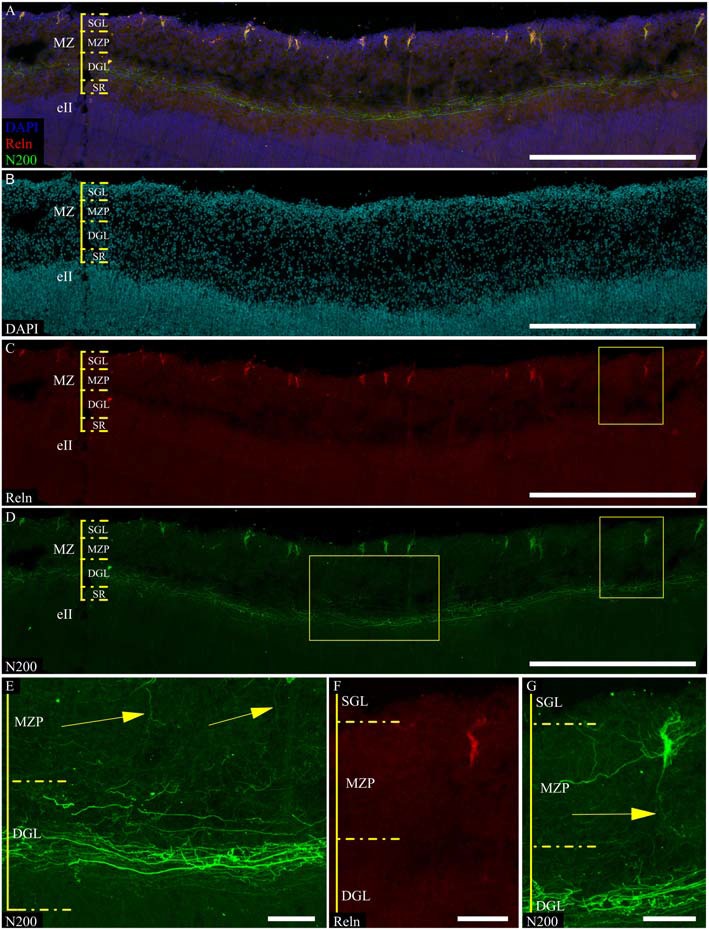
**C–R neurons in the MZ fetal postcentral gyrus at GW 24. (A–D,F,G)** C–R cells stained for N200 using monoclonal N200 antibody (green), Reelin (red), DAPI (blue **(A)**, cyan **(B)**, frozen section. Boxes show the position of the area given at a higher magnification in **(E–G)**. **(E)** N200-positive plexus. Arrows indicate descending C–R cells processes. Scale bars: **(A–D)** 500 μm; **(E–G)** 50 μm.

**Figure 5:**
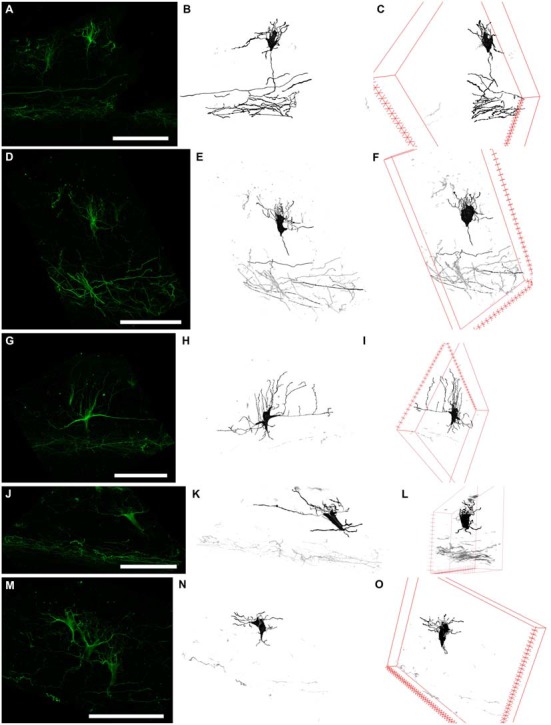
**Three types of N200-positive cells.** Left column **(A,D,G,J,M)** N200-positive C–R cells. Middle column **(B,E,H,K,N)**—C–R cells reconstructed from a confocal stack with a Simple Neurite Tracer. Right column **(C,F,I,L,O)** rotation of the cell in the *x* plane. **(A–C)** and **(D–F)** the first type of C–R cells, **(G–I)**—the second type, **(J–L)** and **(M–O)**—the third type. Scale bars: **(A,D,G,J,M)** 100 μm.

Reelin-positive cells were found throughout the entire MZP. The cell bodies were polymorphic: oval shaped, fusiform, and triangular with vertical and horizontal orientations (Figures 4A,F). The second round of staining revealed N200-positive processes in all of these cells, thereby allowing us to describe three general types of morphology (Figures 4B–D,G, 5). Because we cannot confidently differentiate axons and dendrites, in this publication the thicker processes which determine the shape of neuron hereafter are called stem processes in contrast to the thinner or lateral ones.

The first cell type had a vertically oriented fusiform or pear-like body. The upper pole of the soma gave rise to many unbranched processes ascending to the pial surface. The bottom pole gave rise to a single conical at its foundation and descending stem process, which gives off thin horizontal branches in the course of its descent to plexus (Figures 5A,D). The second cell type had a horizontally oriented triangular or fusiform/oval shaped body and two stem processes, following horizontally from the opposite poles of the cell (length up to 60–70 μm), as well as short ascending and rarely, short descending branches throughout their full length. The short unbranched processes ascended up to the pial surface. One thin descending process starts from the lower pole and rarely traced far from cell body (Figure 5G). The third type had a triangular cell body with an apex looking down. The cell body gave rise to a number unbranched processes ascending towards the pial surface from the side angles of cell horizontally in opposite directions and two long, stretched stem processes, which had multiple thin ascending branches and rarely some short descending branches. The lower pole of the cell gave off a single, well-defined, descending stem process which reached the plexus and formed in its course several thin horizontal branches (Figures 5J,M).

To clarify the morphological types of C–R neurons and their proportion in MZ, a 3D reconstruction of a confocal *z*-stack was made (*n* = 100). Approximately 40% of all cells belong to the first type, 20% to the second type and 40% to the third type. Nevertheless, depending on the angle of rotation of the reconstructed cell in the x-plane, the cell shape and preferential direction of stem processes (horizontal or vertical) changed (Figure 5) which allowed us to conclude that a particular morphotype definition strongly depends on the slice plane. As a result of this analysis, we concluded that all of the C–R cells belong to a single morphotype—a triangular cell body shape with a downwardly facing apex, strong horizontal processes that extend from the upper corners of the body and form a number of vertically ascending branches. Downstream from the lower cell pole, a single descending process extended and gave off along its course some horizontal branches. It then thins towards the plexus.

Because of these results, morphometric measurements were performed on the reconstructed images of the C–R cells in the plane, which allowed us to estimate the maximum area of the soma. We were also taking the depth of the neurons within the MZP into consideration. The analysis showed that all of the C–R cells could be divided into two subpopulations: 75% cells lying directly under the SGL with a medium cell body size (93.4 ± 12.5 μm^2^) and 25% large cells (244.5 ± 34.8 μm^2^, *p* ≤ 0.01) located below in close proximity to the plexus.

### Organization and Localization of the Marginal Zone Plexus

According to a classic conception, the plexus is formed by C–R cell axons. We found that mN200 is an outstanding marker for plexus, which allows us to discern individual fibers. In all of the studied cortical areas with the section plane passing strictly frontally or sagittally, the plexus had a thickness of 50.7 ± 1.71 μm and consisted of tightly packed fibers. Most of these fibers had runs parallel to the pial surface. On the parasagittal sections, the plexus looked looser with its fibers more often following at an angle to each other. The individual fibers could be traced a considerable distance and were divided into two types with significantly different thicknesses (*p* ≤ 0.01): thin 0.7 ± 0.03 μm and thick 1.2 ± 0.13 μm (Figure 4E). It should be noted that the distal portion of C–R cell axons that descended to the plexus were much thinner (0.7 ± 0.09 μm, *p* ≤ 0.01) than the thick fibers of the plexus (Figures 4C,E).

**Figure 6:**
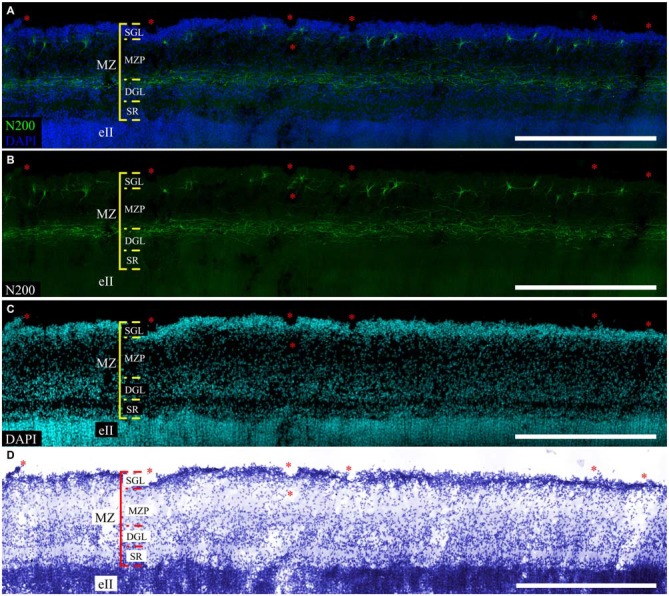
**Localization of the N200-positive C–R cells and plexus in MZ at GW 26. (A–C)**—Plexus and C–R cells stained for N200 with the monoclonal antibody (green), DAPI (blue **(A)**, cyan **(C)**) frozen section. **(D)**—re-staining the same slice with the Nissl method. (*) fiducial points. Scale bars: **(A–D)** 500 μm.

Refinement and comparison of the immunolabeled C–R cells and plexus relative to the MZ cytoarchitectonic sublayers were performed after Nissl re-staining of the same slice. To co-register the immunofluorescence and Nissl images, we selected easy-to-recognize reference points, such as vessels, slice defects and even some large C–R cells visible on Nissl staining. We found a subpopulation of small C–R cells confined to the upper half of the MZP, with only a partial introduction in the SGL. A subpopulation of large C–R neurons was localized in the lower part of the MZP. The plexus was located at level of the DGL (Figure 6).

## Discussion

Prenatal development of human neocortex occurs heterochronically, which has been confirmed by histological, immunocytochemical and MRI research (Poliakov, 1937, 1966; Moore and Guan, 2001; Rakic, 2006, 2009; Rajagopalan et al., 2011; Zhang et al., 2013). Because the MZ is in control of the CP development, we chose areas in which CP neurons differentiate synchronously: the superior temporal, pre- and postcentral (Krasnoshchekova et al., 2010). The MZ of the chosen areas during GW 24–26 have distinct cytoarchitectonic of alternating cell sparse (MZP, SR) and cell dense (SGL, DGL) layers. Kostović et al. (2004) note that this complex organization is transitory (GW 18–28) and that in the course of CP neuron differentiation, the SGL and DGL gradually disappear as the cell density in them decreases.

Our results indicate that the expression of marker proteins shows a high degree of localization: MAP2-positive cells were only found in the SGL, N200 and Reelin-positive neurons in the MZP, N200-positive processes were forming the plexus at the DGL level. MAP2 is predominately expressed in neurons, while its expression in neuroblasts is negligible (Dehmelt and Halpain, 2005). The onset of MAP2 expression in neurons occurs in the stage of dendritic tree formation, morphotype differentiation and cell involvement in interneuronal connections (Bernhardt and Matus, 1984; Sims et al., 1988). At the threshold of the first and second gestational trimester, MAP2-positive cells are found throughout the MZ of a developing human cortex (Honig et al., 1996). Our data indicate that the expression of MAP2 is confined to the SGL cells in the MZ at GW 24–26. The same is known for the MZ of GW 20–24 (Kostović et al., 2004) and GW 21–25 fetuses (Spreafico et al., 1999). The heavy chain neurofilament protein N200 is expressed in more mature neurons (Liu et al., 1994; Bourne and Rosa, 2005). All of the N200-positive neurons we found were in the MZP. In addition, all of them had distinct C–R cell bodies and process branching. We found that all of the N200-positive neurons in the MZ were also positive for Reelin, whereas the MAP2-positive cells lacked Reelin. Thus, the joint use of two immunomarkers allowed us to discern the C–R cells based on their morphotype and neurochemistry (protein expression).

Currently, the majority of studies classify C–R cells by their morphology and position in the MZ. In their research of the human MZ, Cajal and Retzius described four types of neurons with long and short axons based on their form and cell body size (for review, see Gil et al., 2014). Continuing their classical research, three types of C–R cells were defined in the motor cortex of human fetuses with the Golgi impregnation. Two common types were triangular and horizontal cells. The other one is seldom found and an irregular type. Large triangular C–R cells with a triangular and pear-like body are always localized directly under the pial surface. The horizontal C–R cells were localized deeper, at some distance from the pial surface. Their bodies were horizontally elongated and fusiform-like with two thick dendrites radiating from the opposite sides of the cell. The irregular or stellate C–R cells were in the deepest zone in the MZ (Marin-Padilla and Marin-Padilla, 1982; Marín-Padilla, 2015). Other researchers have proposed different classifications with allocation of small horizontal neurons—Cajal cells and triangular (or pear-like) orthogonal to surface—Retzius cells (Meyer et al., 1999). Researchers have proposed that there could be two heterochronically maturating subpopulations of Reelin-producing C–R cells. Aside from C–R cells, they have also isolated Reelin-positive C–R-like cells, which appear later (Meyer et al., 1998). It is thought that SGL could serve as a proliferative zone for C–R-like cells (see Meyer, 2010).

In the current research we identified three C–R morphotypes co-expressing Reelin and N200. Using the 3D reconstruction, we ensured that all of them belonged to single morphotype of triangular C–R cells. It is worth noting that we could not realize exhaustive morphological analysis of C–R cells because the length of their processes could be more than the slice thickness which would lead to incomplete reconstruction. Our data supported the assumption of Marín-Padilla (2015) that there is a single C–R cell morphotype, which, in the gyrificated cortex, can be observed as a number of different projections and therefore, a number of apparent morphotypes. Our research suggests the existence of two populations of C–R cells based on cell size and position in the MZ: small cells, partly penetrating into SGL, and large cells situated near the plexus. The two mentioned populations probably correspond to the C–R and C–R-like cells classified by Meyer and Goffinet (1998).

In the lower third of the MZ, we were able to clearly differentiate the plexus with thin and thick N200-positive fibers. Notwithstanding the Retzius description of the horizontal plexus in his classical study, data on the plexus organization is scarce, which could be due to the specificity of this structure to the primate and human cortex (Huntley and Jones, 1990; Verney and Derer, 1995; Spreafico et al., 1999; Zečević and Rakic, 2001; Cabrera-Socorro et al., 2007; Meyer, 2010). In accordance to Golgi-impregnation studies, it has been agreed that the plexus is organized by C–R cells axons (see Meyer, 2010; Martínez-Cerdeño and Noctor, 2014; Marín-Padilla, 2015). However, Reelin positivity of plexus and C–R cell axons were noted only in some studies. For example, at the lower MZ, probably at the plexus level, some neuropil positivity to Reelin was noted (Cabrera-Socorro et al., 2007). This corresponds with Reelin positivity of C–R cell processes in mouse brains (Derer et al., 2001). Nevertheless, a majority of studies including these data do not confirm Reelin positivity of the plexus in developing human and primate brains (Meyer and Goffinet, 1998; Zečević and Rakic, 2001; Meyer et al., 2002; Deguchi et al., 2003). Immunopositivity of the plexus fibers at GW 20–24 to SMI-31, which is specific marker for axonal neurofilaments, is used as proof that the plexus is formed by axons (Verney and Derer, 1995). Indirect evidence of plexus formation by C–R cell axons is Calbindin and Calretinin immuno-positivity of both the plexus zone and C–R cells (Verney and Derer, 1995; Spreafico et al., 1999). However, individual fibers could not be distinguished separately. The authors describe this diffuse staining as a “bundle” (Verney and Derer, 1995) or “punctum” (Spreafico et al., 1999).

According to the results of current study, the plexus consists of N200-positive fibers of different thicknesses. Combining N200 immunostaining and cytoarchitectonic reveals that the plexus coincides with the cell-dense DGL layer. Despite the fact that cytoarchitectonically, this layer is clearly visible at midgestation in human cortex, its description and neurochemical characteristics of neurons are shown in only one study, which shows that the scattered cells of the DGL express neuronal nuclear antigen NeuN (Kostović et al., 2004). In addition, small Calbindin- and Calretinin-positive neurons, which are embedded in the plexus, could also be DGL cells (Verney and Derer, 1995). Although the authors do not discuss the matter, they express the assumption of the presence of different processes aside from C–R cell axons in the plexus and believe that they could be initiated by CP neurons. According to our data the N200-positive plexus consists of thin and thick fibers with the descending processes of C–R cells, which are traditionally considered axons. They are two times thinner than the thick fibers. This gives us reason to assume that a human fetus plexus could be formed not only by C–R cells processes but also by other cell processes, possibly by DGL cells, which are unique in the human MZ.

Together, our findings indicate that Reelin-positive cells of MZ at GW 24–26 morphologically are a single morphotype of C–R cells being characterized by two horizontal and one ascending stem processes. This approach will allow us to separate C–R cells from other Reelin-producing neurons which appear at later corticogenesis stages. In addition, our findings support the assumption that a plexus could be formed not only by C–R cells axons but also by other cell processes, possibly, by the poorly researched DGL which is only located as a part of the human MZ.

## Author Contributions

EIK, LAT and PAZ designed the research. LAT, PAZ, RAN and EIK collected the data and analyzed the results. RAN collected post-mortem material. LAT, EIK and PAZ wrote the manuscript text. All of the authors reviewed the manuscript.

## Conflict of Interest Statement

The authors declare that the research was conducted in the absence of any commercial or financial relationships that could be construed as a potential conflict of interest.

## Abbreviations

MZ: marginal zone
C–R: Cajal–Retzius
GW: gestational week
SGL: subpial granular layer
MZP: marginal zone proper
SL: stratum lucidum
DGL: deep granular layer
SR: stratum radiatum
GABA: gamma-aminobutyric acid
MAP2: microtubule-associated protein 2
FFPE: formalin fixed paraffin embedded
N200: neurofilament heavy chain protein
Reln: reelin
TBS: tris-buffered saline
DAPI: 4′,6-diamidino-2-phenylindole
GLI: gray level index
CP: cortical plate
SP: subplate.
